# Colloid Adenocarcinoma of the Lung With Concomitant Pulmonary Actinomycosis: A Case Report

**DOI:** 10.1002/rcr2.70133

**Published:** 2025-02-26

**Authors:** Minoru Sugihara, Daiki Goto, Saki Ishiya, Sawako Okamoto, Hirofumi Takenaka, Tetsuo Taniguchi

**Affiliations:** ^1^ Department of Thoracic Surgery Komaki City Hospital Komaki Aichi Japan; ^2^ Department of Respiratory Medicine Komaki City Hospital Komaki Aichi Japan; ^3^ Department of Thoracic Surgery Nagoya University Hospital Nagoya Aichi Japan

**Keywords:** colloid adenocarcinoma, lung cancer, pulmonary actinomycosis

## Abstract

Colloid adenocarcinoma of the lung is a rare subtype of lung adenocarcinoma characterised by the destruction of pre‐existing airspaces caused by mucin produced by the tumour. Pulmonary actinomycosis is a chronic suppurative lung disease caused by *Actinomyces* that is sometimes difficult to differentiate from lung cancer. Here, we report a case of pulmonary colloid adenocarcinoma complicated by pulmonary actinomycosis. A 66‐year‐old male presented with complaints of cough and bloody sputum and was diagnosed with right upper lobe lung adenocarcinoma. During the preoperative waiting period, the lesion rapidly enlarged, prompting an early right upper lobectomy. Histopathological examination confirmed the coexistence of colloidal adenocarcinoma and pulmonary actinomycosis. The coexistence of lung cancer and pulmonary actinomycosis is extremely rare. This case represents an extremely rare coexistence of lung cancer and pulmonary actinomycosis, where a rapidly enlarging lesion was diagnosed through surgical resection.

## Graphical abstract

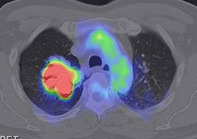

**Abbreviations**
CT: computed tomography
FDG: ^18^F‐fluorodeoxyglucose
PET: positron emission tomography
UICC: Union for International Cancer Control

## 1 Introduction

Colloid adenocarcinoma of the lung is a rare subtype of lung adenocarcinoma, defined as mucin‐producing lung adenocarcinoma that is characterised by the destruction of the surrounding pre‐existing airspaces caused by the abundant extracellular mucin produced by the tumour. Pulmonary actinomycosis is a chronic suppurative lung disease caused by *Actinomyces*, which are anaerobic or microaerophilic gram‐positive, rod‐shaped bacteria. Diagnosis by culture and biopsy is frequently challenging, and surgical resection is required in some cases. Pulmonary actinomycosis can be difficult to differentiate from lung cancers, necessitating careful clinical management. In this report, we present a rare case of colloid adenocarcinoma with concomitant pulmonary actinomycosis, where the lesion rapidly progressed.

## 2 Case Report

**FIGURE 1:**
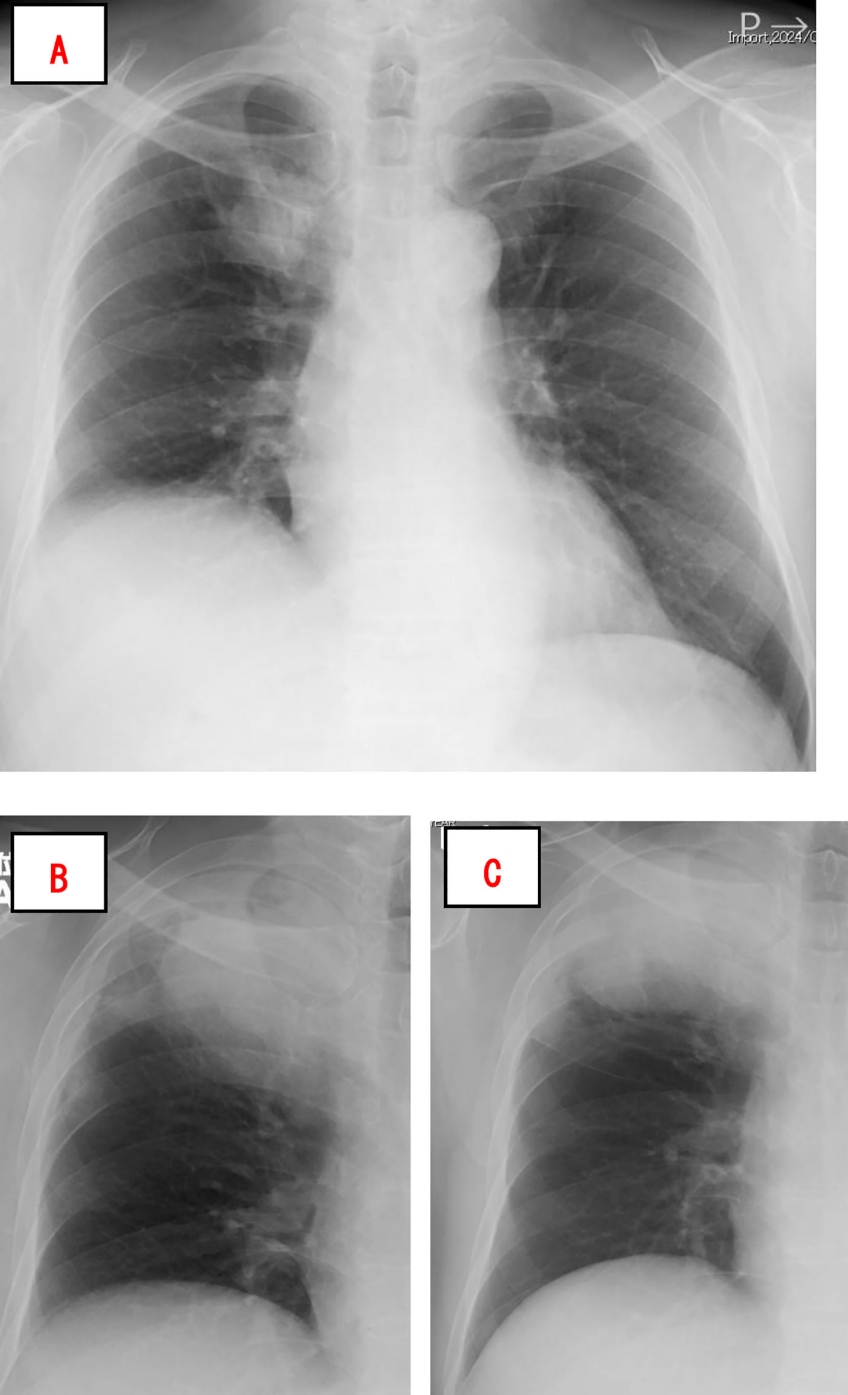
Imaging findings. Chest radiography performed at the initial visit revealed a mass in the right upper lung field (A). Chest radiograph on admission, 1 month after the initial radiograph, showing enlargement of the mass in the right upper lung field (B). Chest radiograph obtained 14 days after antibiotic therapy showing no improvement in the imaging findings (C).

**FIGURE 2:**
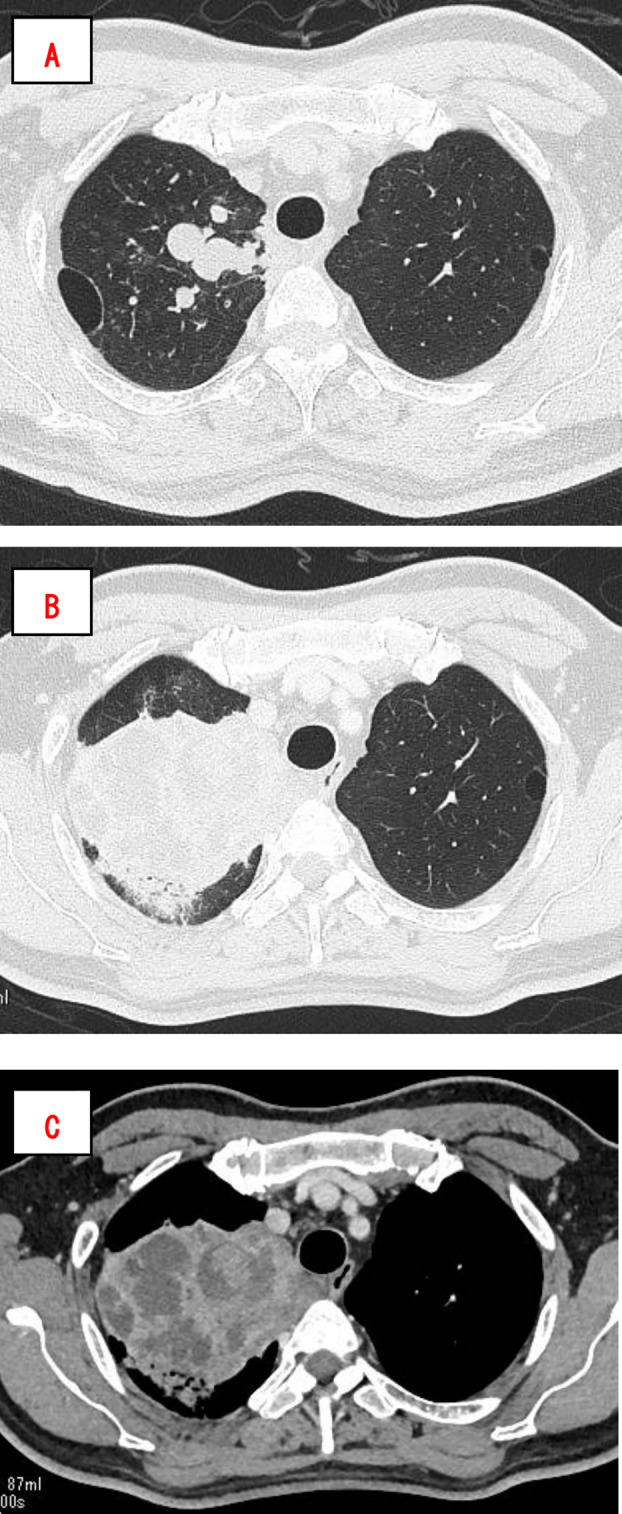
Imaging findings. CT showed a lobulated mass in the right upper lobe, with a maximum diameter of 41 mm (A). CT performed 1 month after the initial CT scan revealed that the tumour was filled with soft tissue density areas and had enlarged, accompanied by peripheral consolidation suggestive of obstructive pneumonia (B, C). CT, computed tomography.

**FIGURE 3:**
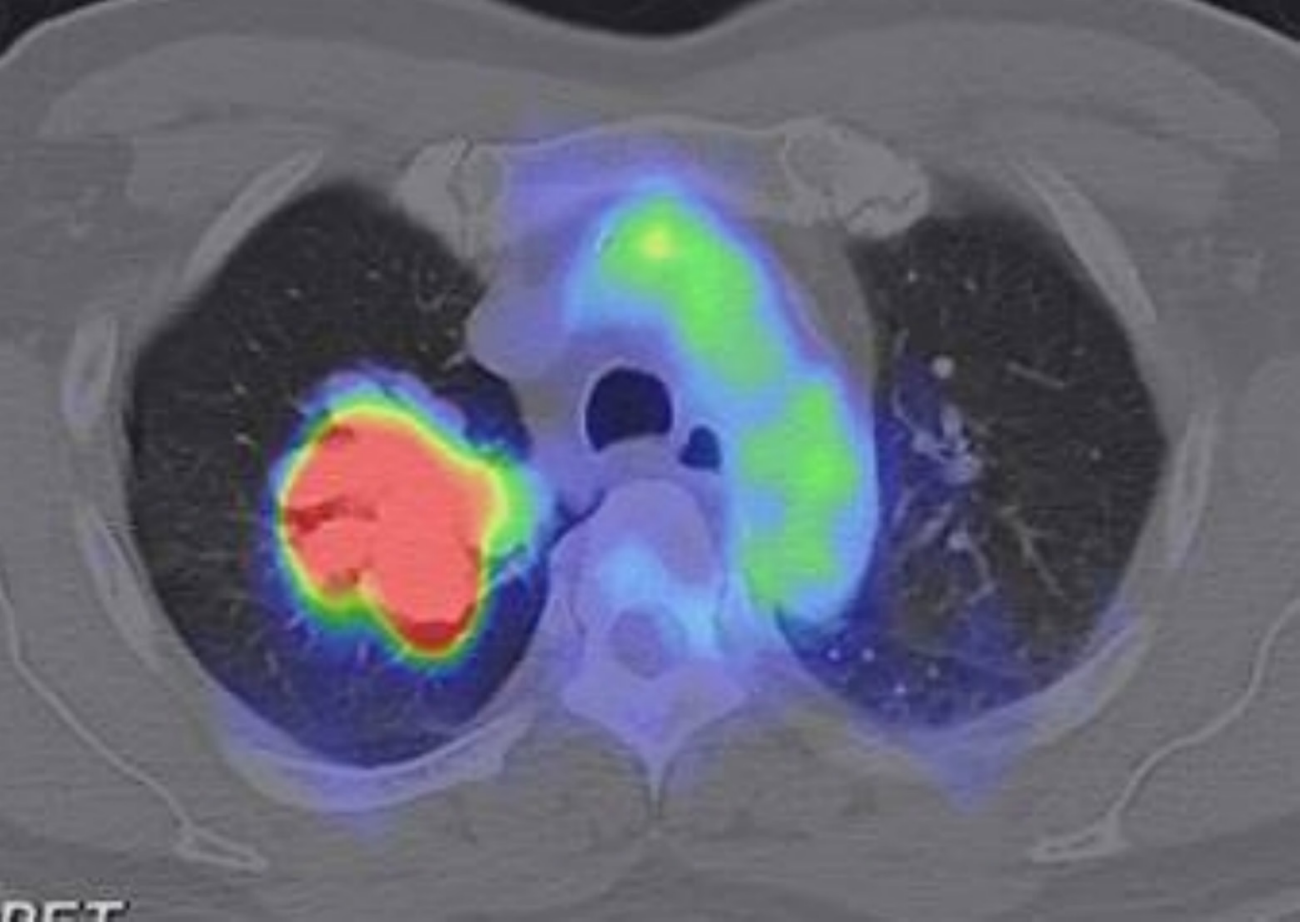
Imaging findings. PET‐CT showed an FDG‐avid mass in the right upper lobe with a maximum standardised uptake value of 12.8. CT, computed tomography; PET, positron emission tomography; FDG, ^18^F‐fluorodeoxyglucose.

The patient was a 66‐year‐old male with a smoking history of 40 packs/year. He presented with a 1‐month history of cough and reported bloody sputum 1 week before the consultation. Chest radiography revealed a mass in the right upper lung field (Figure 1A). Chest computed tomography (CT) revealed a lobulated mass in the right upper lobe with a maximum diameter of 41 mm (Figure 2A). Positron emission tomography (PET)‐CT showed an ^18^F‐fluorodeoxyglucose (FDG)‐avid mass in the right upper lobe with a maximum standardised uptake value of 12.8 (Figure 3). PET‐CT also revealed a slightly FDG‐avid right hilar lymph node but showed no evidence of distant metastasis. Contrast‐enhanced magnetic resonance imaging of the brain showed no evidence of brain metastasis. Bronchoscopy revealed occlusion of the right B1 bronchus by the tumour, and transbronchial biopsy confirmed the diagnosis of lung adenocarcinoma. The patient was diagnosed with right upper lobe lung adenocarcinoma (cT2bN1M0, cStage IIB, Union for International Cancer Control [UICC] 8th edition), and a right upper lobectomy was planned.

During the preoperative waiting period of 1 month, the patient had a fever and returned for further evaluation. Chest CT performed 1 month after the initial scan revealed that the tumour was filled with soft tissue density areas and had enlarged; this was accompanied by peripheral consolidation, suggestive of obstructive pneumonia (Figure 2B,C). Antibiotic therapy was initiated; however, imaging findings did not improve (Figure 1B,C). An early right upper lobectomy with lymph node dissection (ND2a‐1) was performed. The patient's postoperative recovery was uneventful, and he was discharged.

**FIGURE 4:**
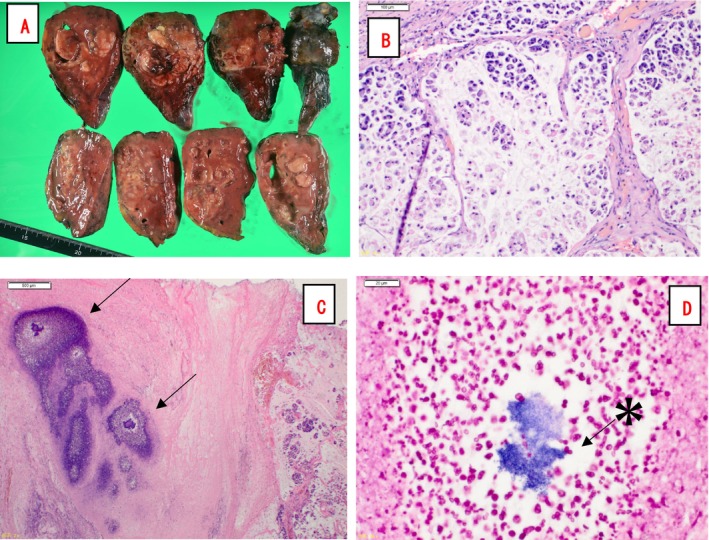
Pathological findings. The resected specimen revealed a mucinous tumour with necrotic‐like areas (A). Tumour nests floating in mucin‐filled cystic spaces with the destruction of pre‐existing lung architecture were observed (B). Actinomycotic colonies (arrows) were identified within the abscessed portions of the tumour (C). Gram staining revealed gram‐positive *Actinomyces* (asterisk) surrounded by inflammatory cells (D).

Pathological examination of the resected specimen revealed a mucinous tumour with necrosis‐like areas (Figure 4A). Histopathological analysis showed tumour nests floating in mucin‐filled cystic spaces with destruction of the pre‐existing lung architecture (Figure 4B), confirming the diagnosis of colloid adenocarcinoma. The maximum tumour diameter and invasive diameter were both 5.5 cm, with no lymph node metastasis, leading to a final pathological staging of pT3N0M0, pStage IIB (UICC 8th edition). Additionally, actinomycotic colonies were identified within the abscessed portions of the tumour (Figure 4C,D), indicating concomitant pulmonary actinomycosis. Since the lung tissue affected by *Actinomyces* was completely resected, no additional treatment for pulmonary actinomycosis was administered, and adjuvant chemotherapy was initiated.

## 3 Discussion

Colloid adenocarcinoma of the lungs was first defined as a rare subtype in the 2015 World Health Organisation classification. It is characterised by the destruction of the surrounding pre‐existing air spaces due to the abundant extracellular mucin produced by the tumour. Histologically, differentiating it from invasive mucinous adenocarcinoma (IMA) can be challenging, and alveolar architecture destruction is a key diagnostic feature. Accounting for only approximately 0.13% of primary lung cancers, its clinical characteristics remain poorly understood, although they are generally considered to progress slowly.

On CT, colloid lung adenocarcinoma typically presents as a well‐circumscribed solitary lesion with low‐attenuation areas reflecting intratumoral mucin; these findings can help differentiate it from IMA, which is characterised by consolidation. PET‐CT frequently shows low FDG uptake with mild uptake along the tumour wall or septa. Owing to its high mucin content and low malignant cell density, a biopsy diagnosis is often difficult, necessitating surgical resection for a definitive diagnosis. Genetic mutations in EGFR or ALK are rare, and KRAS mutations and the presence of non‐colloidal components have been associated with poor prognosis [1, 2].

Pulmonary actinomycosis is a chronic suppurative lung disease caused by *Actinomyces*, which are anaerobic or microaerophilic gram‐positive, rod‐shaped bacteria; it is often associated with underlying conditions such as dental caries or chronic obstructive pulmonary disease. On CT, it typically presents as consolidation or a mass‐like opacity, sometimes with central low‐attenuation areas or cavitation, reflecting abscess formation or necrosis. Diagnosis using nonsurgical procedures is usually challenging because of the difficulty of bacterial culture and the presence of granulomatous tissue; consequently, surgical resection is frequently required for a definitive diagnosis. Treatment comprises penicillin‐based antibiotics, generally administered over a prolonged period of 6–12 months. Surgical intervention is considered in cases refractory to antibiotics or when symptoms such as hemoptysis are present. No established consensus exists on the necessity of antibiotic therapy following surgical resection for pulmonary actinomycosis. In this case, postoperative antibiotic treatment was not administered, as the affected lung tissue was considered to be completely resected. Distinguishing this condition from lung cancer is particularly challenging and requires careful management [3].

The coexistence of lung cancer and pulmonary actinomycosis at the same site is extremely rare, with only two cases reported in the literature [3]. Both cases were initially diagnosed with pulmonary actinomycosis and were treated with antibiotics without improvement, leading to a subsequent diagnosis of lung cancer upon reevaluation.

Previous studies have suggested a potential link between infection and the progression of lung cancer; reports have indicated that chronic inflammation caused by pulmonary tuberculosis may promote carcinogenesis [4] or that T‐cell activation induced by bacterial infection may accelerate tumour growth [5]. In the present case, the coexistence of colloid adenocarcinoma and pulmonary actinomycosis was confirmed. In this case, the initial tumour growth rate was slow, and the pathological findings revealed only a small abscessed area. Therefore, the primary cause of lesion enlargement is considered to be tumour progression. Although it is difficult to conclude definitively, the possibility that *Actinomyces* infection contributed to the rapid tumour growth cannot be ruled out.

Colloid adenocarcinoma is extremely rare, and its coexistence with pulmonary actinomycosis at the same site is even more unusual. This case represents a rare coexistence of these two conditions, where the lesion rapidly enlarged and was diagnosed through surgical resection.

## Author Contributions

M.S. wrote the manuscript. M.S. and T.T. performed surgical procedures. All the authors have discussed the contents of the manuscript. T.T. supervised manuscript editing. All the authors have read and approved the final version of the manuscript.

## Ethics Statement

The authors declare that appropriate written informed consent was obtained for the publication of this manuscript and the accompanying images.

## Conflicts of Interest

The authors declare no conflicts of interest.

## Data Availability

Data sharing not applicable to this article as no datasetswere generated or analysed during the current study.
